
JOURNAL INFORMATION
==============================
NLM Title Abbreviation: J Diabetes Investig
Journal ID: J Diabetes Investig
NLM Journal ID: 101520702
Journal ID: JDI

Journal of Diabetes Investigation

ISSN: 2040-1116
EISSN: 2040-1124
Publisher: Wiley

ARTICLE INFORMATION
==============================
PMCID: PMC4906259
DOI: 10.1111/jdi.12510
Article ID: JDI12510
Article version: 1
Subjects: Author Index; Abstracts from The 7th Scientific Meeting of the Asian Association for the Study of Diabetes and the Annual Scientific Meeting of the Hong Kong Society of Endocrinology, Metabolism and Reproduction, 21‐22 November 2015, Hong Kong, China; Author Index

Author Index

Electronic publication date: 2016 Jun 14
Print publication date: 2016 May
Volume: 7
Issue: Suppl 2
Issue title: Abstracts from The 7th Scientific Meeting of the Asian Association for the Study of Diabetes and the Annual Scientific Meeting of the Hong Kong Society of Endocrinology, Metabolism and Reproduction, 21‐22 November 2015, Hong Kong, China
First page: 56
Last page: 59
Copyright: © 2016 The Authors. Journal of Diabetes Investigation published by Asian Association for the Study of Diabetes (AASD) and John Wiley & Sons Australia, Ltd
License: This is an open access article under the terms of the Creative Commons Attribution License, which permits use, distribution and reproduction in any medium, provided the original work is properly cited.
License URL: https://creativecommons.org/licenses/by/4.0/

Page count: 4
source-schema-version-number: 2.0
component-id: jdi12510
cover-date: May 2016
details-of-publishers-convertor: Converter:WILEY_ML3GV2_TO_NLMPMC version:4.9.1 mode:remove_FC converted:14.06.2016

==============================
JDI Journal of Diabetes Investigation Open access
Official Journal of the Asian Association for the Study of Diabetes

### Author Index

A
Abe, M., 21, 24
Adachi, H., 50
Ahn, S., 49
Ahn, YH., 41
Akamizu, T., 27, 49
Akasu, S., 52
Amakura, Y., 37
Amo-Shinoki, K., 30
Ando, T., 43, 43
Ang, Y., 45
Antoku, S., 21
Aoki, M., 24
Arakawa, S., 31
Araki, E., 24, 35, 35
Arimura, E., 24
Ariyasu, H., 49
Asada, Y., 15, 32
Asahara, S-I., 36
Asano, E., 43, 43
Au Yeung, YC., 32

B
Bao, Y., 11, 12, 12, 16, 16, 27, 30, 38, 39, 40
Benesch, C., 33
Bochmann, L., 33
Bunoy, L., 21
Byun, D-W., 33

C
Carol, CLH., 31
Chan, JC-N., 10
Chan, JCN., 42
Chan, KS., 47
Chan, LW., 46, 47, 51
Chan, RWS., 51
Chan, TF., 42
Chan, WS., 47
Chan, YKD., 48
Chang, E., 23
Chen, H., 16
Chen, M., 13
Chen, S., 40
Cheung, CY., 41
Chida, D., 20
Chikazawa, G., 25
Chikazawa, S., 29
Ching, JH., 45
Ching, KM., 47
Chiu, PC., 54, 54
Chiu, PCN., 53, 53, 53
Choi, CH., 32
Choi, SH., 44, 49, 51
Choi, WH., 41
Chow, W., 53
Chow, W-S., 41
Chung, CT., 41
Chung, HY., 25, 40
Chung, T., 47
Clado-Reyes, C., 12, 39
Costigan, T., 36

D
Daikoku, H., 14
Daimon, M., 29
Deeg, M., 36
Dezaki, K., 30
Doi, A., 49
Duan, R., 29

E
Ebisu, T., 34
Edralin, D., 29
Endo, K., 31, 33
Enomoto, T., 48

F
Fan, H., 41
Fang, L., 11
Fang, Q., 39
Feng, B., 40
Filippova, E., 29
Fong, C., 53
Fong, CH., 41
Fujii, H., 12, 14, 14, 15, 32, 50
Fujikawa, J., 52
Fujimoto, W., 9
Fujine, S., 49
Fujiwara, Y., 36
Fukuda, K., 24
Fukunaga, T., 51
Funato, M., 27
Fung, LM., 48
Furusawa, C., 36
Furuta, H., 27, 49
Fushimi, N., 39

G
Gangwani, R., 41
Gheni, G., 37
Ghorbani, A., 55
Ghosh, S., 45
Goto, Y., 52
Guo, K., 16
Guo, L., 17
Guo, X., 17
Guo, Y., 53

H
Hachiya, H., 39
Haensler, J., 33
Hamaguchi, T., 26, 46
Hamamoto, Y., 34, 52
Hamasaki, A., 34, 47, 52
Hamasaki, H., 50
Hamasaki, S., 44
Han, JH., 29
Han, S-Y., 40
Hao, Y., 11, 12, 12, 16, 27
Hara, M., 36
Harada, N., 47
Harada, T., 47
Hasebe, M., 25
Hasegawa, M., 50
Hashim, M., 37
Hata, M., 42
Hatami, A., 55
Hatanaka, M., 30
Hayakawa, T., 48
Hayami, T., 43
Hayashi, Y., 43
Hayashida, K., 31
He, M., 26
He, R., 33
He, Z., 15
Heise, T., 33
Heng, BH., 45
Himeno, T., 42, 43, 43
Hiradate, E., 49
Hirai, N., 43
Hiraishi, C., 51
Hiraoka, A., 25
Hirasawa, Y., 34, 35
Hirawsawa, Y., 44
Hirose, N., 44
Hirose, T., 18
Hirota, Y., 26, 46
Hirukawa, H., 25
Hishino, Y., 31, 33
Honjo, S., 34, 52
Horiuchi, M., 24
Hoseyni, SJ., 55
Hoshikawa, R., 37
Hosseini, A., 55
Hotta, A., 35
Hou, X., 30, 39
Hsieh, CJ., 13
Hu, C., 13, 13, 14, 15, 20, 30
Hu, J., 40
Hu, X., 12, 27
Hu, Y., 38
Hu, Y., 33
Hua, X., 38
Hui, EY., 41
Hui, Z., 11
Huster, W., 36
Hwang, W-M., 40
Hyo, T., 44

I
Ihara, Y., 36
Iimura, T., 28

© 2016 The Authors. Journal of Diabetes Investigation published by Asian Association for the Study of Diabetes (AASD) and John Wiley & Sons Australia, Ltd
This is an open access article under the terms of the Creative Commons Attribution License, which permits use, distribution and reproduction in any medium, provided the original work is properly cited

Ilag, L., 36
Inagaki, N., 16, 27, 47
Ishida, H., 35, 35
Ishida, M., 15
Ishii, N., 24
Ishikawa, S-e., 22
Ito, H., 21
Ito, K., 22, 30
Ito, S., 14, 39
Ito, T., 48
Iwasaki, K., 47
Iwasaki, M., 44
Iwata, S., 52

**J**
Jang, HC., 44, 49, 51
Jia, W., 11, 12, 12, 13, 13, 14, 15, 16, 16, 17, 20, 27, 30, 38, 38, 39, 40
Jia, Weiping., 33
Jia, WP., 37
Jian, L., 31
Jiang, F., 14, 15
Jiang, S., 39
Joo, E., 47
Jugueta, R., 21
Jun, Z., 11
Jung, CH., 23
Jung, KY., 50
Jungong, Z., 11
Junling, T., 11
Junxi, L., 11

**K**
Kabeya, Y., 17, 17
Kakei, M., 22, 30
Kaku, K., 25
Kamiya, H., 42, 43, 43
Kanba, A., 29
Kaneto, H., 25
Kanno, A., 36
Kanno, K., 14
Kasagi, R., 43
Kato, K., 17, 43
Kato, M., 14, 22
Kato, N., 14, 22
Kato, Y., 43, 43
Katsuki, T., 17, 17
Katsuya, D., 22
Katsuyama, H., 50
Kawada, Y., 36
Kawagoe, Y., 14, 14, 15, 50
kawagoe, Y., 32
Kawaguchi, A., 46, 50
Kawai, H., 39
Kawai, M., 39
Kawakami, K., 18
Kawakami, M., 22, 30
Kawano, S., 52
Kawasaki, Y., 34, 52
Kawashima, H., 49
Kido, Y., 36
Kim, C., 44
Kim, D-M., 44
Kim, DS., 40, 41
Kim, DW., 20
Kim, H-J., 33
Kim, HM., 20
Kim, H-S., 25
Kim, KM., 44, 49, 51
Kim, S-H., 25, 40
Kim, Y., 23
Kim, YJ., 49
Kimura, T., 25
Kimura-Koyanagi, M., 36
Kinoshita, H., 24
Kirinoki-Ichikawa, S., 28
Kiriyama, K., 25
Kitaoka, M., 35, 35
Kitaoka, T., 14
Kitatani, N., 44
Ko, WKW., 26
Kobayashi, M., 50
Kodani, E., 14
Koga, S., 11
Kohara, K., 25
Kojima, C., 43, 43
Komada, H., 26, 46
Komiyama, M., 43
Kondo, H., 14
Kondo, M., 25, 42, 43, 43
Kondo, T., 46, 48
Kondo, Y., 27
Konrad, R., 36
Kosuga, M., 49
Koto, J., 28, 32, 34
Kovalik, JP., 45
Ku, EJ., 50
Kubota, N., 9
Kubota, Y., 28
Kunisada, M., 14
Kurisu, S., 27
Kurose, T., 34, 44
Kusaba, M., 35
Kusuki, K., 25
Kuwabara, R., 24
Kuwata, H., 43
Kwak, M-K., 33
Kwok, KH., 41

**L**
Lam, K., 10, 53
Lam, KKW., 53, 53
Lam, KS., 41
Lam, PKE., 48
Lau, CS., 49
Lau, YFE., 41
Lee, C., 41, 53
Lee, C-L., 53, 54, 54, 55
Lee, CL., 54
Lee, D-H., 49
Lee, H., 47
Lee, HM., 42
Lee, JW., 51
Lee, K-F., 53
Lee, MY., 52
Lee, PC., 41
Lee, WJ., 23
Leung, JYY., 48
Lew, (Presenter Only) T., 29, 29
Li, H., 39
Li, JW., 42
Li, L., 38
Li, Lianxi., 33
Li, P., 29
Li, Q., 17, 40
Li, RH., 54
Li, W., 31
Ligen, A., 11
Lim, D-M., 40
Lim, S., 44, 49, 52
Lim, SC., 45
Lim-Teodoro, A., 12, 21, 39
Lin, C-W., 22
Liu, F., 38, 38, 38, 40
Liu, Fang., 33
Liu, JJ., 45
Liu, M-Y., 22, 25
Low, SKM., 45
Lu, J., 16, 30, 39
Lu, W., 40
Lu, Z., 27
Luo, Y., 11, 12, 12, 16, 27

**M**
Ma, A., 26
Ma, CW., 49
Ma, RCW., 42
Ma, V., 50
Ma, X., 11, 12, 12, 16, 27
Maejima, K., 37
Makita, K., 29
Mao, D., 49
Marchianti, A., 24
Marumo, K., 31
Maruyama, Y., 31
Mastronardi, CA., 19
Masui, Y., 46
Matsubara, T., 42
Matsuda, T., 36
Matsuhashi, Y., 29
Matsui, S., 27
Matsuki, K., 29
Matsuki, R., 34, 35
Matsumoto, K., 28, 32, 34
Matsumura, T., 24
Matsuno, S., 49
Matsuoka, A., 26, 46
Mei, S., 53, 53
Menya, T., 44
Mifune, M., 21
Mine, Y., 24
Mishima, S., 50
Mitsutsuka, T., 48

© 2016 The Authors. Journal of Diabetes Investigation published by
Asian Association for the Study of Diabetes (AASD) and John Wiley & Sons Australia, Ltd

Miyabe, M., 42
Miyakawa, T., 12, 14, 14, 15, 32, 50
Miyamoto, M., 28
Miyao, M., 25
Miyawaki, M., 49
Miyazaki, Y., 15
Mizuno, Y., 25
Mizunuma, A., 31
Mo, Y., 17
Mojarad, M., 55
Moon, JH., 44, 44, 49
Mori, A., 39
Mori, M., 31
Mori, T., 15, 32
Morishita, Y., 30
Morita, H., 54
Morita, M., 14
Moriyama, S., 51
Motegi, M., 43
Mu, KD., 37
Murakami, H., 29
Murakami, M., 54
Murakami, S., 24
Muraoka, A., 47

**N**

Nagai, M., 37
Nagai, R., 24, 31, 36, 37
Nagao, E., 43, 43
Naito, E., 43, 43
Nakagami, T., 31
Nakai, H., 43, 43
Nakakuma, M., 24
Nakamura, J., 42, 43, 43
Nakamura, N., 42
Nakamura, T., 26, 46
Nakanishi, I., 27
Nakayama, H., 52
Nam, JY., 20
Nanasawa, H., 48
Nanjo, K., 27, 49
Naruse, K., 42
Neeman, T., 19
Ng, CM., 32
Ng, EHY., 51, 53
Ng, SS., 51
Ng, YW., 32
Ning, G., 29
Nishi, M., 27, 49
Nishiyama, H., 20
Nisimura, K., 15
Nisio, S., 21
Niu, Z., 52
Noda, H., 44
Noda, S., 43, 43
Nogawa, M., 15
Nogawa, M., 12, 14, 14, 32, 50
Nohara, M., 52
Nojiri, R., 31
Nonaka, A., 15, 32
Nonogaki, K., 48
Norio, H., 16
Numata, Y., 31
Nunes, VS., 19

**O**

O, K., 20
Obara, M., 49
Obata, A., 25
Ogawa, K-I., 27
Ogawa, R., 28
Ogawa, W., 26, 36, 46
Oh, H., 40, 41
Oh, TJ., 51
Ohashi, N., 39
Ohno, R-I., 24, 37
Ohta, A., 27
Oikawa, Y., 17, 17
Okada, Y., 46
Okamoto, T., 44
Okamura, E., 34, 52
Okauchi, S., 25
Okuno, M., 24
Omi, M., 42
Omoto, T., 21
Ong, CN., 45
Onizaki, S., 43
Ookawara, S., 30
Oota, M., 31
Osafune, K., 27
Ota, T., 32
Otaka, H., 29
Owatari, R., 36

**P**

pan, P., 16
Pan, X., 11, 12, 12, 27
Pang, C., 38
Pang, RT., 52
Park, C-Y., 23
Park, H-K., 33
Park, JH., 40, 41
Park, J-Y., 23
Park, KH., 20
Park, KS., 49, 49
Park, K-Y., 40
Paulino, H., 39
Paz-Filho, G., 19
Pei, X., 46, 51
Pei, XM., 50
Peng, D., 20
Pilong, W., 24
Pollom, R., 36
Poudyal, H., 16
Prince, M., 36
Pura, G., 12

**R**

Rodriguez, A., 29
Rodriguez, AJ., 19
Roh, E., 44
Ronald, D., 49
Rudd, JA., 46
Ruiz, LN., 29

**S**

S, A., 48
Saigusa, M., 37
Saito, H., 11
Saito, M., 31
Sakaguchi, K., 26, 46
Sako, A., 46, 48, 50
Salimo, E., 12
Sarna, L., 20
Sasaki, D., 28, 32, 34
Sasaki, H., 27
Sata, A., 25
Sato, R., 14
Satogami, M., 43
Satoh, J., 35, 35
Seino, S., 36, 37
Seino, Y., 34, 35, 35, 44
Sekine, A., 32
Senokuchi, T., 24
Sham, P-C., 41
Shen, Y., 11
Shibue, K., 47
Shibuya, T., 39
Shihara, N., 35, 35
Shimada, A., 17, 17
Shimauchi, R., 43
Shimizu, I., 25
Shimizu, S., 44
Shimizu, W., 28
Shimoda, M., 25
Shinozaki, M., 21
Shirakawa, J-i., 31
Shiu, SWM., 43
Sid, V., 20
Sin, TK., 46
Siow, YL., 20
Siu, MFP., 46
Siu, PM., 46, 47, 50, 51
Soga, R., 52
Song, KH., 29
Song, SO., 20
Song, YD., 20
Sou, A., 46
Subramaniam, T., 45
Sudo, T., 27
Suematsu, N., 26, 46
Sugawa, H., 37
Sugawara, K., 49
Sugiura, Y., 36, 43, 43
Suh, K-I., 33
Sum, CF., 45
Suzuki, J., 24
Suzuki, K., 47
Suzuki, Y., 18, 18, 19, 19, 21, 23, 26, 28, 38, 44

**T**

Tabei, K., 30
Tai, ES., 45
Tajiri, Y., 52
Takagi, G., 28
Takagi, M., 43
Takahashi, A., 20

© 2016 The Authors. Journal of Diabetes Investigation published by Asian Association for the Study of Diabetes (AASD) and John Wiley & Sons Australia, Ltd

Takahashi, H., 49
Takahashi, K., 29
Takahashi, R., 49
Takamura, H., 14
Takanashi, Y., 15
Takayama, C., 15, 32
Takazawa, K., 11
Takeda, K., 15
Takeishi, S., 39
Takesue, M., 14
Tam, BT., 46
Tam, HKV., 48
Tam, WH., 49
Tamura, H., 49
Tamura, K., 25
Tan, KCB., 43
Tanabe, K., 30
Tanaka, H., 27
Tanaka, N., 43
Tang, J., 12
Tanizawa, Y., 30
Tatsumi, T., 15
Terauchi, Y., 35, 35
Tezuka, A., 28
Tian, H., 17
Tiu, SC., 32
Togane, M., 21
Tokumoto, S., 34, 52
Tomita, M., 17, 17
Tomonaga, O., 31
Toyoda, T., 27
Tsun, JGS., 43
Tsuruta, M., 52
Tsurutani, Y., 25
Tu, M-Y., 22, 25

**U**
Uchida, J., 17, 17
Ueki, A., 14
Uematsu, Y., 31
Ugwu, FN., 50
Uju, Y., 48
Umemoto, Y., 34, 35
Uose, K., 43
Urai, S., 46
Urakami, T., 24
Uraki, S., 49
Ushikubo, E., 31

**V**
Vijayan, M., 54

**W**
Wada, N., 52
Wada, Y., 52
Wakabayashi, C., 36
Wang, C., 37
Wang, CC., 49
Wang, CR., 37
Wang, J., 13
Wang, T., 14
Wang, X., 51, 55
Wang, Y., 53
Watadani, R., 28, 32, 34
Watanabe, A., 27
Watanabe, K., 43
Watanabe, S., 43
Watanabe, Y., 12, 14, 14, 15, 32, 50
Weiping, J., 11
Wong, AOL., 26
Wong, CL., 48
Wong, CS., 46, 47, 51
Wong, C-W., 53
Wong, DS., 41
Wong, RL., 41
Wong, SC., 47
Wong, TW., 47
Wong, Y., 43
Woo, Y., 53
Woo, Y-C., 41
Wu, G., 39
Wu, N., 20

**X**
Xiao, Y., 12, 12
Xiaoyan, Z., 11
Xiong, Q., 12, 16
Xu, A., 39, 41, 53
Xu, S., 51
Xu, W., 40
Xu, Y., 12, 16

**Y**
Yabe, D., 34, 35, 35, 44
Yada, T., 22, 30
Yagi, T., 15
Yamada, H., 22, 30
Yamada, K., 52
Yamada, T., 25
Yamada, Y., 35, 35, 43, 43
Yamaguchi, E., 34, 52
Yamaguchi, K., 43
Yamamoto, H., 35
Yamanaka, M., 24, 37
Yamane, S., 47
Yamaneki, M., 27
Yan, D., 13
Yanagimachi, M., 29
Yanai, H., 46, 48, 50
Yang, H., 17
Yang, J., 29
Yau, WY., 47
Yeh, J., 36
Yeung, C., 53
Yeung, WS., 52
Yeung, WSB., 51, 53, 53, 53
Yimin, C., 11
Yin, DC., 37
Ying, MT., 46
Yip, KY., 42
Yip, SP., 46, 46
Yokoi, N., 37
Yoo, HJ., 40
Yoo, M-H., 33
Yoshida, M., 22, 30, 43
Yoshida, N., 14
Yoshida, Y., 31, 39
Yoshimura, H., 50
Yoshimura, M., 37
Yoshino, G., 18
Yoshino, H., 18
Yoshinobu, S., 52
Yoshitaka, H., 25
Yu, AP., 46, 50
Yu, H., 16, 31
Yu, JM., 40
Yu, PHA., 47
Yu, S., 47
Yu, SH., 25, 40
Yu, TX., 31
Yuen, CK., 52
Yuen, M., 53
Yuen, MM., 41
Yueng, W., 41
Yumura, T., 39
Yun, LJ., 31
Yung, BY., 46, 46, 51
Yuqian, B., 11

**Z**
Zeng, H., 38
Zeng, Hui., 33
Zhang, J., 39
Zhang, L., 16, 30, 39
Zhang, M., 17
Zhang, R., 13, 14, 15, 20
Zhao, F., 16
Zhao, J., 30, 38, 38
Zhao, W., 38, 40
Zhao, Y., 37
Zheng, F., 17
Zhou, J., 17
Zhu, J., 16
Zhu, W., 52
Zhuang, X., 27
Zielonka, J., 36

© 2016 The Authors. Journal of Diabetes Investigation published by
Asian Association for the Study of Diabetes (AASD) and John Wiley & Sons Australia, Ltd
